# The association between Fc gamma RIIb expression levels and chronic hepatitis B virus infection progression

**DOI:** 10.1186/s12879-021-06918-7

**Published:** 2021-12-08

**Authors:** Jinglan Jin, Yuwei Liu, Xiaotong Xu, Zhongfeng Wang, Junqi Niu

**Affiliations:** Department of Hepatology, First Bethune Hospital of Jilin University, 71 Xin Min Street, Changchun, Jilin, 130021 People’s Republic of China

**Keywords:** FcγRIIb, HBV infection, Chronic hepatitis B

## Abstract

**Background:**

Fc gamma receptor IIb (FcγRIIb) is an important inhibitory receptor that plays vital roles in regulating various immune response processes and the pathogenesis of many infectious diseases. The purpose of our research was to evaluate FcγRIIb expression in serum and liver biopsy specimens from hepatitis B virus (HBV)-infected patients and to explore the association of FcγRIIb with chronic HBV infection.

**Methods:**

Enzyme-linked immunosorbent assay (ELISA) was adopted to measure the serum FcγRIIb levels in 119 HBV-infected patients and 24 healthy controls. An immunohistochemical method was then employed to identify FcγRIIb expression in biopsy specimens from patients with chronic hepatitis B (CHB). The integrated optical density (IOD) value was measured to represent FcγRIIb expression levels.

**Results:**

Serum FcγRIIb levels were decreased in CHB patients compared to controls (P < 0.001). The FcγRIIb levels in the CHB patient group were remarkably lower than those in the HBV carrier group (P < 0.001). In addition, FcγRIIb levels were negatively associated with AST and ALT (r = −0.3936, P = 0.0063; r = −0.3459, P = 0.0097, respectively). The IOD values of FcγRIIb expression in the moderate and severe CHB groups were significantly lower than those in the control group (P = 0.006 and P < 0.001, respectively). The FcγRIIb level tended to be lower with pathological changes related to hepatitis. Furthermore, correlation analysis revealed that FcγRIIb had negative correlations with AST and ALT (r = −0.688, P = 0.0016; r = −0.686, P = 0.0017, respectively) but a positive association with the platelet count (r = 0.6464, P = 0.0038).

**Conclusions:**

FcγRIIb levels are significantly related to chronic HBV infection and the progression of CHB. Changes in FcγRIIb may affect the progression of liver inflammation and fibrosis in CHB patients.

## Background

Chronic hepatitis B virus (HBV) infection has always been a major public health challenge worldwide. According to the World Health Organization (WHO) global hepatitis report, the number of HBV cases in 2015 was 257 million, and the infection rate was 3.5% [1]. Chronic HBV infection is a primary cause of liver cirrhosis and hepatocellular carcinoma (HCC). Moreover, it accounts for 786,000 HBV-associated deaths annually, making it the tenth major cause of mortality worldwide [2]. HBV can stimulate both innate and adaptive immune responses. Suppression of adaptive immunity is known to be a crucial factor in maintaining persistent HBV infection through inhibition of the killing effect of CD8+ T cells [3, 4]. The innate immune response has been confirmed to be involved in viral clearance, and interferons, key innate immune effector molecules, have long been used to treat HBV infections [5].

Fc gamma receptor IIb (FcγRIIb) is the sole inhibitory Fc gamma receptor (FcγR) and can negatively modulate downstream signalling pathways [6]. It has three common isoforms, b1, b2 and b3, and b3 is the only soluble isoform that lacks a transmembrane domain and the first cytoplasmic domain [7, 8]. FcγRIIb is expressed on the surface of nearly all leukocytes, including B cells, and is involved in regulating cell-mediated immunity [9, 10]. A recent study showed that FcγRIIb can also be expressed in memory CD8+ T cells and regulate their activation and survival [11]. Another prominent feature of FcγRIIb is that it can be expressed on nonhaematopoietic cells, such as airway smooth muscle cells [12] and liver sinusoidal endothelial cells (LESCs) [13]. FcγRIIb expressed in LESCs is responsible for the removal of small immunocomplexes in hepatic sinusoids [14], which is essential for sustaining liver immune homeostasis. Ishikawa et al. conducted research on nonalcoholic fatty liver disease biopsy samples and found that the progression of liver inflammation and fibrosis was associated with decreased expression of FcγRIIb in LSECs and might influence the scavenger functions of this receptor [15]. A study focused on HCC discovered that decreased expression of FcγRIIb was more likely to indicate a more advanced cancer grade [16]. Moreover, fibrinogen-like protein 2 (FGL2) is a member of the fibrinogen superfamily and is secreted by regulatory T (Treg) cells [17]. It has been reported to be capable of binding to FcγRIIb and exerting an immunosuppressive effect [18].

FcγRIIb is a regulatory molecule involved in various processes in both innate and adaptive immunity, such as antigen presentation, FcγR-mediated cellular activation, and apoptosis. It also regulates various signalling pathways, such as the TLR [19] and MAPK pathways [20]. FcγRIIb is an important checkpoint molecule involved in humoral tolerance in the human immune system. FcγRIIb hypofunctioning may result in activation of immune response signalling and viral clearance [17]. Liver injury occurrence in HBV infection is strongly dependent on the host immune responses to the virus and is often accompanied by elevations in transaminase levels. Thus, FcγRIIb might be pivotal in immune reactions involving an interaction between HBV and the host. Moreover, as a ligand of FcγRIIb, FGL2 participates in the immune response to persistent viral infections by regulating the FcγRIIb immune-inhibitory pathway. FGL2 expression has been associated with HBV infection and related to the clinical outcomes of HBV-associated liver diseases [21]. This study measured FcγRIIb expression levels in HBV patients and analysed correlations with clinical parameters in an attempt to elucidate the role of FcγRIIb in the immune response process of chronic HBV infection.

## Methods

### Patients used for serum sample collection

Serum samples were obtained from 119 chronic HBV infected patients and 24 healthy individuals at the First Hospital of Jilin University between June 2019 and August 2020. The inclusion criteria for the chronic HBV-infected patients included the following: (1) hepatitis B surface antigen (HBsAg)+  > 6 months, (2) no antiviral therapy, and (3) no liver imaging showing cirrhosis or tumour. All the healthy controls were recruited from the medical examination centre, and they were not found to have any diseases after physical examination. Patients who had other liver diseases, such as alcoholic hepatitis, fatty liver disease, acute or chronic infectious diseases, or autoimmune diseases, were excluded. According to the Guidelines for the Prevention and Treatment of Chronic Hepatitis B (2019 Edition) issued by the Chinese Medical Association [22], the patients were divided into HBV carriers with persistent normal alanine transaminase (ALT)/aspartate aminotransferase (AST) levels and chronic hepatitis B (CHB) patients whose ALT/AST levels were persistently or intermittently elevated. The basic and clinical data of the patients and healthy controls are presented in Table 1. The study was approved by the research ethics committee of the First Hospital of Jilin University.

**Table 1:** Characteristics of patients with chronic HBV infection

Characteristics	Health control	HBV carrier	Chronic hepatitis B	*P* value
Patients	24	64	55	
Age				
Mean ± SD	46.0 ± 12.3	41.2 ± 11.8	40.6 ± 11.4	0.143
Media (Range)	44 (23–67)	41 (16–65)	41 (18–64)	0.183
Gender (%)				
Male	10 (41.7%)	38 (59.4%)	39 (70.9%)	0.047
Female	14 (58.3%)	26 (40.6%)	16 (29.1%)	
HBeAg (%)^a^				
Positive		22 (34.4%)	38 (74.5%)	< 0.001
Negative		42 (65.6%)	13 (25.5%)	
HBV-DNA (log10 copies/ml)		4.4 ± 2.5	6.1 ± 1.9	< 0.001
Mean ± SD				
ALT (U/L)		22.9 (16.1–35.5)	91.7 (68.1–184.1)	< 0.001
Media (Range)				
AST (U/L)		23.1 (19.8–31.0)	70.1 (45.9–103.8)	< 0.001
Media (Range)				
γ-GT (U/L)		17.8 (12.3–27.8)	50.0 (26.2–82.3)	< 0.001
Median (Range)				
ALP 9U/L)		68.5 (56.6–81.8)	72.2 (60.3–89.7)	< 0.001
Media (Range)				
Albumin (g/L)		44.9 (42.1–46.5)	41.7 (39.9–44.6)	< 0.001
Mean ± SD				

HBeAg, hepatitis B e antigen; ALT, alanine amino transferase; AST, aspartate amino transferase; γ-GT, γ-glutamyl transpeptidase; ALP, alkaline phosphatase

^a^Data for 4 patients were not available

### Patients used for liver tissue sample collection

Liver tissue samples were obtained from 12 patients with CHB and 4 controls with normal hepatic tissue who had undergone liver biopsy at the First Hospital of Jilin University between May 2015 and December 2018. Pathological diagnoses were classified into stages according to the Scheuer scoring system (G0-4 and S0-4) [23]. The grouping criteria were derived from the “Viral hepatitis prevention and treatment program” issued by the Chinese Society of Infectious Diseases, Chinese Medical Association (2001 Edition). Mild hepatitis B was defined as pathological stage G1 ~ 2, S0-2; moderate hepatitis B was defined as G3, S1 ~ 3; and severe hepatitis B was defined as G4, S2-4 [24]. Fasting blood samples were collected from the patients before liver biopsy. The basic and clinical data of all patients are presented in Table 2. The study was approved by the ethical review board of the First Hospital of Jilin University.

**Table 2:** Characteristics of patients with chronic hepatitis B and controls who underwent liver biopsy

Diagnosis	Age	Gender	Biological parameters						Pathological stage	
			AST (U/L)	ALT (U/L)	ALP (U/L)	γ-GT(U/L)	ALB (g/L)	PLT (10^9/L)	G	S
Severe CHB	37	Male	179	278	183	183	31.9	174	4	3
Severe CHB	32	Female	131	99	75	34	42.1	234	4	4
Severe CHB	42	Female	121	116	42	15	35.9	113	4	3
Severe CHB	49	Male	115.9	87.2	52.3	94.6	43	84	4	4
Severe CHB	52	Male	158.9	193.1	146.7	106.3	29.4	131	4	4
Moderate CHB	35	Female	26.1	19.7	53.4	21.1	39.8	186	3	3
Moderate CHB	47	Female	37.3	54.4	72.4	25.6	36.2	187	3	1
Moderate CHB	19	Male	45	56	117.3	34.2	42.1	147	3	2
Moderate CHB	23	Male	113.8	94.5	130.1	74.8	46.4	171	3	3
Mild CHB	30	Male	24.9	39.8	77.4	30.3	42.3	237	1	1
Mild CHB	35	Male	32.4	31	69.6	24.6	45.8	133	2	2
Mild CHB	35	Male	44.6	55.2	97.5	27.9	46.8	177	2	1
Mild CHB	49	Male	28.8	64.7	109.7	18.6	35	181	2	1
Mild CHB	37	Male	22.2	33.3	64.9	14.4	37.2	187	1	1
Control	46	Male	29.1	32.8	94.5	17.9	42.8	219	0	0
Control	54	Female	15.4	9.9	82.4	161.3	40.2	347	0	1
Control	19	Male	49.1	44.9	46	17.1	49.3	246	0	0
Control	54	Female	32.7	39.2	114.4	88.4	44.5	232	0	0

ALT, alanine amino transferase; AST, aspartate amino transferase; ALP, alkaline phosphataseγ; GT, γ-glutamyl transpeptidase; ALB, albumin; PLT platelet counting; G, inflammatory grade; S, fibrosis stage

### Enzyme-linked immunosorbent assay (ELISA)

ELISA experiments (soluble FcγRIIb: Jingmei, Jiangsu, China) were conducted according to the manufacturers’ protocols. Briefly, serum samples were incubated in 96-well plates coated with primary antibodies. After an incubation, the plate was washed, and horseradish peroxidase-labelled secondary antibodies were added to each well of the plate. Then, after the incubation period, the plate was washed again. Immunocomplexes were detected with secondary antibody-specific development reagents. The optical density of each well was read with a microplate reader (Multiskan Sky, Thermo Fisher, USA).

### Immunohistochemistry and morphometry

Immunohistochemical staining for FcγRIIb was performed on liver tissue specimens. Paraffin sections were dewaxed in water. Antigen unmasking was performed with 10 mM citric buffering solution (pH 6.0) for ten minutes at 95 °C. Then, 3% hydrogen peroxide was added to each section to block the activity of endogenous peroxidases. After washing with PBS, the sections were incubated with primary rabbit anti-human FcγRIIb antiserum (Abcam, USA). After washing with PBS, the sections were incubated with an enzyme-conjugated anti-rabbit secondary antibody. The staining was detected with diaminobenzidine, and then the sections were counterstained with haematoxylin. The integrated optical density (IOD) values of the sample sections in every group were determined via Image-Pro Plus 6.0 software, with the tissue imaging program used after the collection of the sample images under a light microscope (40×). Five fields of vision were selected stochastically to measure the positive IOD values, and then the average of the IOD values, which was deemed to represent the relative expression of FcγRIIb, was calculated.

### Basic biochemical parameters

Basic biochemical parameters were evaluated with common approaches. Herein, our study analysed the serum concentrations of AST, ALT, alkaline phosphatase (ALP), gamma-glutamyl transpeptidase (γ-GTP), albumin (ALB), HBV-DNA, etc. The HBV-DNA results were converted to log 10 IU/ml.

### Statistics

All statistical analyses were performed with GraphPad Prism 8. Experimental data are expressed as the mean ± standard deviation (SD). Comparisons of FcγRIIb expression levels were performed by the nonparametric Kruskal–Wallis test. Comparisons of the IOD value for FcγRIIb expression were performed by one-way ANOVA. Correlations were calculated using Spearman correlation analysis. A two-tailed p-value < 0.05 was considered statistically significant.

## Result

### Serum expression levels of FcγRIIb in chronic HBV-infected patients

#### Participate characteristics

Table 1 summarized the information of participates enrolled in this study including data for age, gender and clinical parameters. In total, most of participates were men. No differences in age existed between two groups. In the HBV carrier group, there were more patients positive for hepatitis B e antigen (HBeAg). In the CHB group, more patients were negative for HBeAg but positive for anti- HBeAg antibodies.

### Serum FcγRIIb expression level

**Fig. 1:**
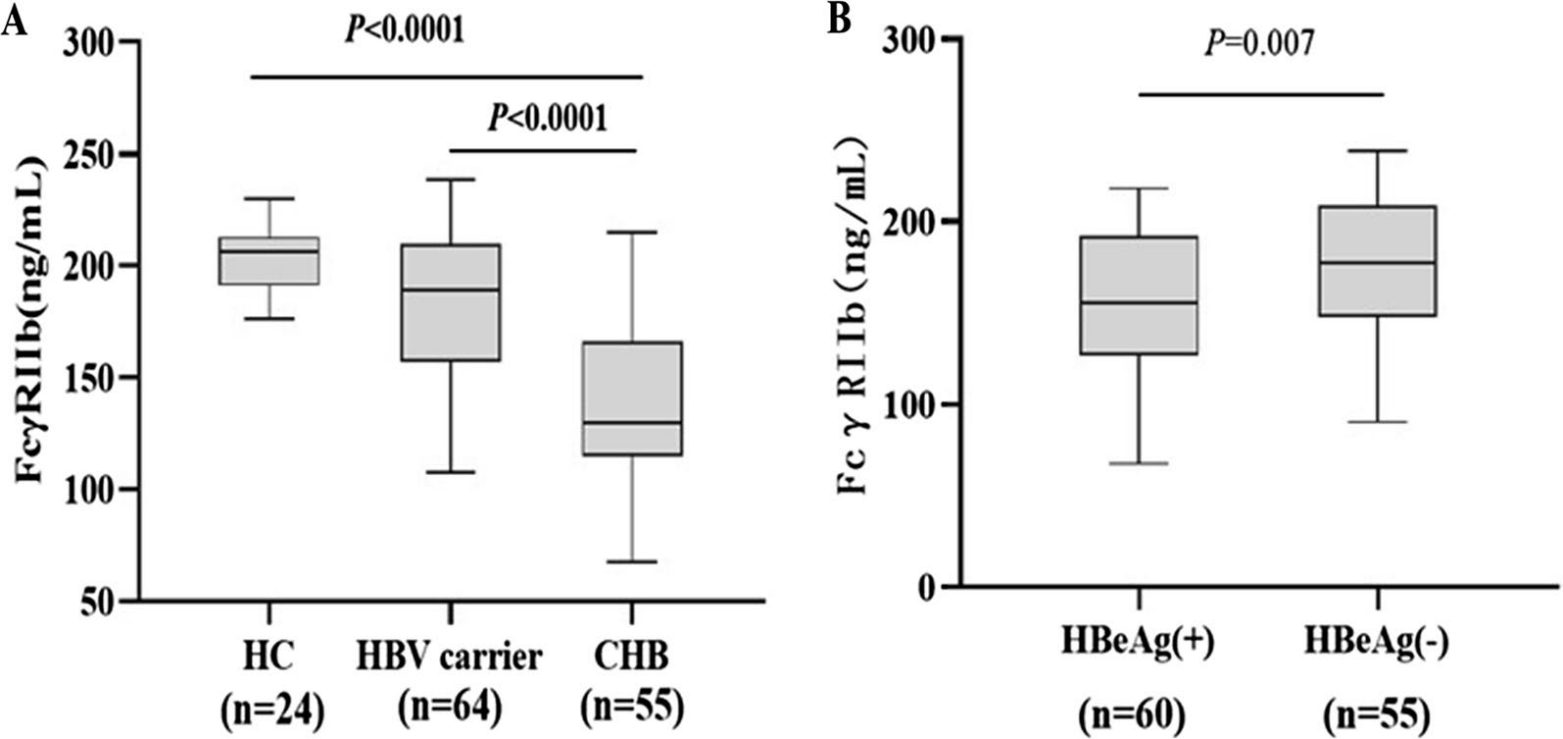
FcγRIIb levels in patients with chronic HBV infection and in healthy controls. **A** FcγRIIb levels were measured in study subjects and compared among subgroups. **B** Comparison of the HBeAg positive group and HBeAg negative group. HC, healthy control; CHB, chronic hepatitis B. Box plots illustrate the medians and interquartile range. P values were calculated by the Mann–Whitney-Wilcoxon test

The values of serum FcγRIIb were 201.9 ± 15.19 ng/ml in the healthy control group, 183.9 ± 33.47 ng/ml in the HBV carrier group and 141.0 ± 37.14 ng/ml in the CHB group. Compared with the healthy control group, the HBV carrier group showed no significant difference in the serum FcγRIIb level (*P* = 0.123), while the CHB patient group exhibited a significantly lower FcγRIIb level (*P* < 0.001) (Fig. 1A). The serum FcγRIIb level in the CHB patient group was significantly lower than in the HBV carrier group (*P* < 0.001). Among all the HBV infected patients, the serum FcγRIIb level of HBeAg positive patients was statistically significantly lower than that of HBeAg negative patients(*P* = 0.007) (Fig. 1B).

### Correlations with biochemical parameters

**Fig. 2:**
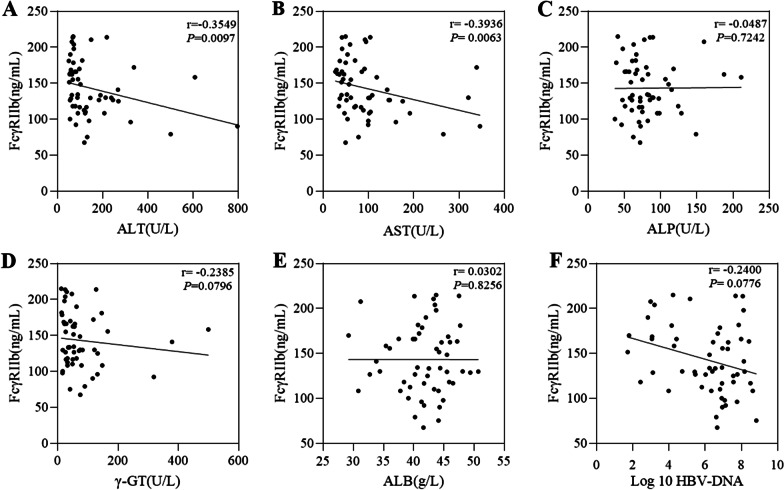
Correlations of FcγRIIb levels with clinical parameters of HBV infection. The correlations of FcγRIIb levels with different available clinical parameters were calculated by using Spearman’s rank correlation coefficient test. The Spearman’s rho and P value are also presented. Correlation **A** FcγRIIb levels and alanine amino transferase (ALT); **B** FcγRIIb levels and aspartate amino transferase (AST); **C** FcγRIIb levels and alkaline phosphatase; **D** FcγRIIb levels and γ-glutamyl transpeptidase (γ-GT) levels; **E** FcγRIIb levels and albumin levels and **F** FcγRIIb levels and the HBV-DNA load

The correlations of FcγRIIb expression levels with serum biochemical parameters in CHB patients are shown in Fig. 2. FcγRIIb level had a negative correlation with AST (r = −0.3936, *P* = 0.0063) and ALT (r = −0.3459, *P* = 0.0097). The regressive lines of serum ALP, γ-GTP and HBV DNA had negative slopes, but no statistically significant association existed. The regression line for ALB had a positive slope, although no significant correlation was found.

### Expression levels of FcγRIIb in tissue from CHB patients

#### Patient characteristics

Table 2 summarized the information of 18 participants enrolled in the study, including data for age, gender and clinical parameters. Based on biopsy proven pathologically inspection, five patients were confirmed to have severe CHB, four patients were confirmed to have moderate CHB, and five patients were confirmed to have mild CHB. Four patients with normal liver tissue were included in the control group.

### FcγRIIb expression level in liver tissue

**Fig. 3:**
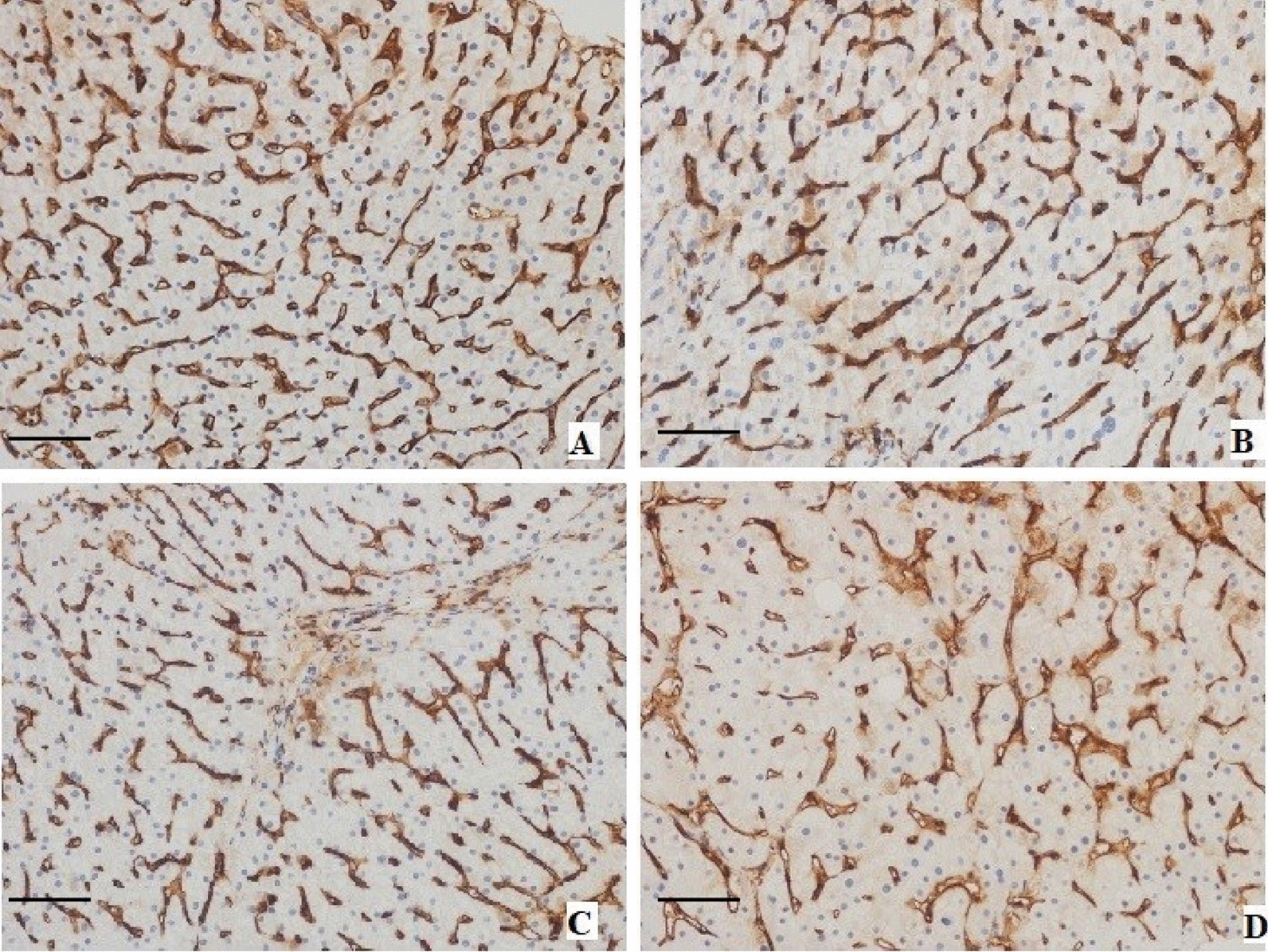
Representative light microscopy images of FcγRIIb expression in liver tissue samples from patients with chronic hepatitis B and controls (**a**: controls; **b**: mild CHB; **c**: moderate CHB; **d**: severe CHB). FcγRIIb signals were detected with DAB (brown) Nuclei were counterstained with haematoxylin (blue). Scale bars represent 100 μm

**Fig. 4:**
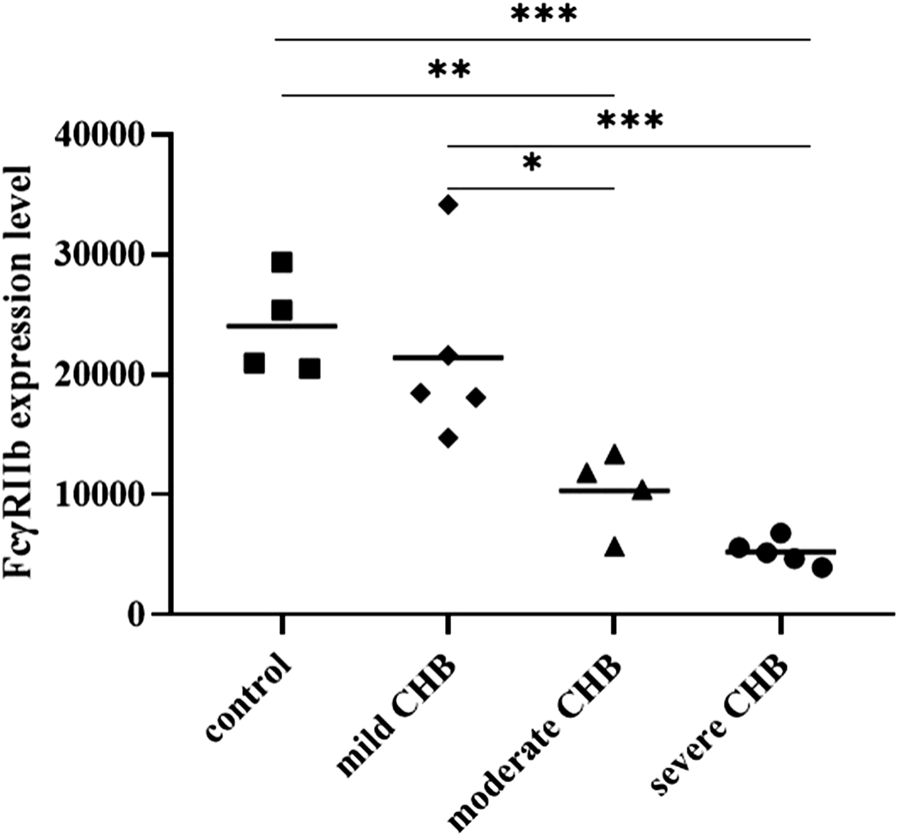
FcγRIIb levels in liver tissue samples from patients with chronic HBV infection and healthy controls. P values were calculated by one-way ANOVA

Typical optical microscope images for patients in different group are presented in Fig. 3. The FcγRIIb signals were discovered in only LSECs [13]. In the study, the IOD values of FcγRIIb were 23696.08 ± 3847.33 in control group, 21392.93 ± 7536.20 in the mild CHB group, 10287.25 ± 2878.82 in the moderate CHB group and 5214.78 ± 1071.27 in the severe CHB group. Compared with control group, the mild CHB group showed no significant difference in the FcγRIIb level, while the FcγRIIb levels in the moderate CHB and severe CHB groups were significantly lower than those in the control group (*P* = 0.006, *P* < 0.001, respectively). The FcγRIIb expression levels in the severe CHB and moderate CHB group were also lower than in the mild CHB group (*P* < 0.001, *P* = 0.018, respectively). In other words, the FcγRIIb level tended to decrease with the pathological changes related to hepatitis (Fig. 4).

### Correlation with biochemical parameters and pathological stage

The correlations of the FcγRIIb expression level in liver biopsy specimens with biochemical parameters and pathological stage are shown Table 3. A correlation analysis showed that FcγRIIb had negative relationships with AST (r = −0.688, *P* = 0.0016) and ALT (r = −0.686, *P* = 0.0017), and a positive relationship with platelets (r = 0.6464, *P* = 0.0038). The regression lines for serum ALP, γ-GTP and HBV-DNA presented negative slopes, but no significant associations existed. The regression line for ALB had a positive slope, although there was no significant correlation. Evaluation of the pathological stage revealed that FcγRIIb had significantly negative correlations with inflammation grade (r = −0.913, *P* < 0.001) and fibrosis stage (r = −0.875, *P* < 0.001). It could be inferred that the decline in FcγRIIb levels in the development of chronic hepatitis was related to worsening of liver inflammation and fibrosis (Table 3).

**Table 3:** Correlations of FcγRIIb levels with clinical parameters of HBV infected patients

	FcγRIIB expression level	
	r	*P*
AST	−0.69	0.00
ALT	−0.69	0.00
ALP	0.06	0.81
γ- GT	−0.20	0.44
ALB	0.45	0.06
PTL	0.65	0.00
Fibrosis Grades	−0.91	< 0.001
Inflammatory Stage	−0.88	< 0.001

The correlations of FcγRIIb levels with different available clinical parameters were calculated by using Spearman’s rank correlation coefficient test. The Spearman’s rho and P values are presented

### Predictive value of FcγRIIb levels for the liver inflammation grade

**Fig. 5:**
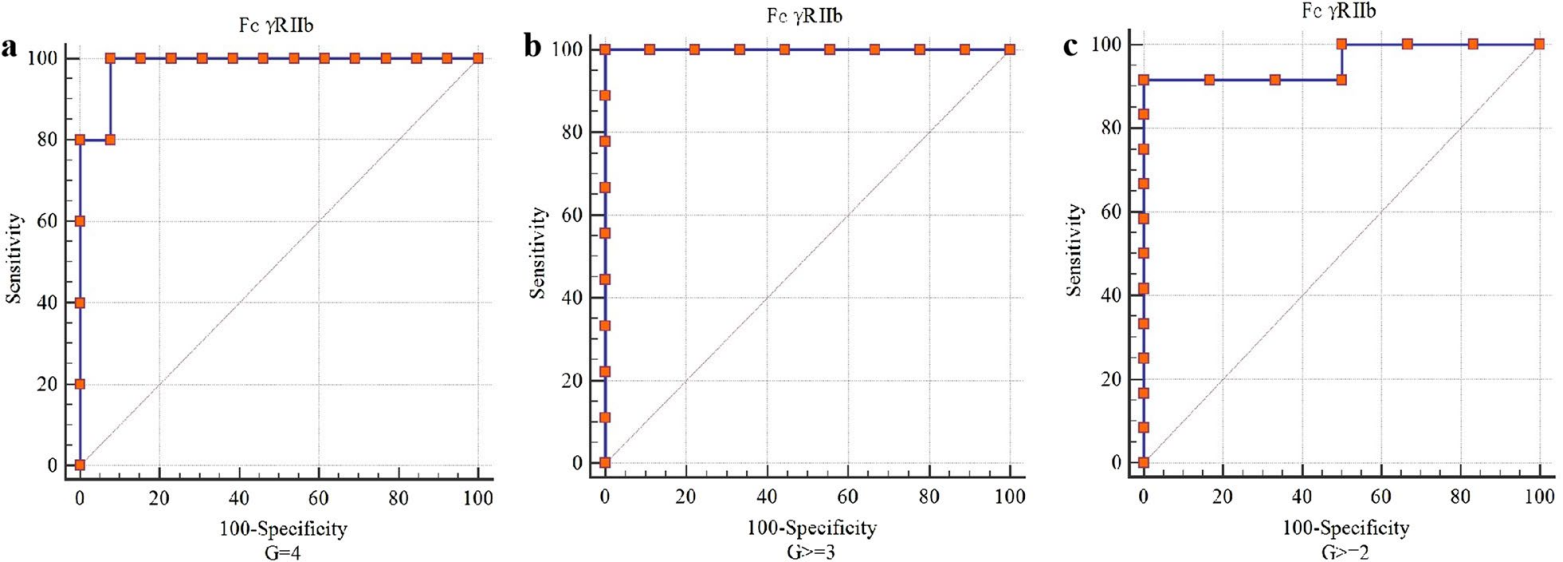
ROC curve for FcγRIIb expression levels predicting liver inflammation grade. ROC, receiver operating curve

For diagnosing G = 4 liver inflammation, the areas under the receiving operator characteristic curves (AUROCs) indicated that the predictive value of FcγRIIb levels was 0.98 (95% confidence interval (CI) 0.79–1.00), the sensitivity was 1.00, and the specificity was 0.92. For diagnosing G ≥ 3 liver inflammation, the AUROCs indicated that the predictive value of FcγRIIb levels was 1.00 (95% CI 0.82–1.00), the sensitivity was 1.00, and the specificity was 1.00. For diagnosing G ≥ 2 liver inflammation, the AUROCs indicated that the predictive value of FcγRIIb levels was 0.95 (95% CI 0.75–1.00), the sensitivity was 0.92, and the specificity was 1.00 (Fig. 5).

### Predictive value of FcγRIIb levels for the liver fibrosis stage

**Fig. 6:**
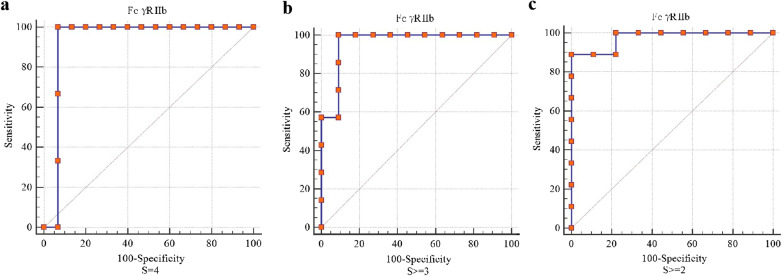
ROC curve of FcγRIIb expression levels predicting liver fibrosis stage. ROC, receiver operating curve

For diagnosing S = 4 liver fibrosis, the AUROCs indicated that the predictive value of FcγRIIb levels was 0.93 (95% CI 0.71–0.99), the sensitivity was 1.00, and the specificity was 0.93. For diagnosing S ≥ 3 liver fibrosis, the AUROCs indicated that the predictive value of FcγRIIb levels was 0.96 (95% CI 0.75–1.00), the sensitivity was 1.00, and the specificity was 0.98. For diagnosing S ≥ 2 liver fibrosis, the AUROCs indicated that the predictive value of FcγRIIb levels was 0.95 (95% CI 0.77–1.00), the sensitivity was 0.89, and the specificity was 1.00 (Fig. 6).

## Discussion

FcγRIIb is an inhibitory receptor that functions as a regulation molecule in the immune system and is vital in the progression of various autoimmune diseases and infectious diseases. In our previous study, we performed qRT-PCR assay to assess FcγRIIb mRNA expression in HBV infected patients [25]. The study showed that FcγRIIb expression was higher in the HBV carrier group than in the CHB group. In addition, we speculate that during the immune tolerance stage of HBV infection, FcγRIIb expression played an immunosuppressive role. Thus, declining FcγRIIb expressions in patients with CHB leads to decreasing inhibition of the immune response and results in host immune activation. In this study, we discovered that the FcγRIIb levels of serum samples from CHB patients were significantly decreased compared to those of samples from HBV carriers and healthy controls. The study further suggested that FcγRIIb could be a potential target of the immune response during chronic HBV infection.

During the early stage of virus infection, the innate immunity suppresses replicative activities and transmission, whereas the adaptive immunity mainly affects the virus removal during the late of infection period [26]. HBV can be recognized by related receptors and trigger antiviral innate immunity. Then the envelope antigen and nucleocapsid antigen of the virus can stimulate the adaptive immune response. With the help of CD4+ T cell, antibodies can be produced and combine with HBV to form an immunocomplex that can be phagocytized by monocytes and macrophages. FcγRIIb can regulate expression and function of immune cells in innate immunity, such as maturation and antigen presentation ability of dendritic cells and the phagocytic ability of monocytes and macrophages [6]. A study has shown HBsAg-specific B cells in CHB patients exhibited a rised expression of suppressive receptors like PD-1 and FcγRIIb to suppress B cell activation and anti-viral immunity [27]. The clearance of persistent HBV infection relies on the cytotoxic immune response of HBV-specificity CD8+ T cell. CD8+ T cells can induce cytotoxic reactions by recognizing antigen presented by MHC-I on antigen-presenting cells (APCs), which can promote the apoptosis of HBV infected hepatocytes [28]. However, the persistent HBV infection may result from HBV-specific CD8+ T cells functional impairment; in other words, the majority of HBV-specificity CD8+ T cells are able to undergo activation but exhibit poor proliferation and undergo functional exhausted [29, 30]. The mechanism of this functional decline is very complex; for example, the expression of inhibitory receptors and relevant ligands, such as PD-1 and PD-L1, on hepatocytes is increased [31, 32]. FcγRIIb can help dendrite cells acquire antigen from immunocomplexes and present antigens to Ag-specificity CD4+ and CD8+ T cell to induce robust immune reactions [27]. The HBV vaccine chimegen is a chimera immunotherapy protein for treating CHB, and it is targeted dendritic cell. It could bind to immature dendrite cells and promote internalization of FcγRII and mannose receptor (CD206) by endocytosis, which can lead to elevated MHC I and MHC II surface expression, induce T cell proliferation and restore HBV specific T cell function [33]. Treg cells are specific subgroup of CD4+ T cells which are crucial for establishing and maintaining immunotolerance. Tregs from CHB patients can suppress the HBV specificity T cell immune response and result in a chronic, persistent HBV infection [34]. Moreover, FcγRIIb can bind to FGL2, working as an effector molecule of Treg cells, and has immunoregulatory activity [35]. Hoang and his colleagues discovered that FGL2 levels were remarkably increased in patients with cirrhosis and HCC compared to controls [21]. Therefore, FcγRIIb is vital for immune responses and is involved in the pathogenesistic mechanism of virus infections, especially that of HBV infection.

The levels of AST and ALT could denote the severity of liver damage during HBV infection, and elevated liver enzyme levels can be viewed as biomarkers of immune stimulation. In the early clinical studies, researchers found that patients with elevated ALT values had a higher rate of HBV clearance than patients with normal ALT values. This result highlights the necessity of the activating the host immune system against viral antigens [36]. In our study, the FcγRIIb levels in the CHB patient group were significantly lower than in the HBV carrier group and had negative relationships with ALT and AST. Immune tolerance occurs frequently during chronic HBV infection. Therefore, FcγRIIb may be a checkpoint molecule that can be exploited to break the immune tolerance state, restore effector T cells, clear infected hepatocytes, and eliminate circulating immune antigens by humoral immunity.

FcγRIIb is expressed on LSECs, and the liver is the major site of small immune complex, including blood bore SIC clearance [13, 14]. FcγRIIb is a kind of scavenger receptor and the distributional feature of FcγRIIb has been presented in intrahepatic lobules in healthy livers [37]. Some studies have revealed an association between FcγRIIb expression change in LESCs and liver diseases. Ishikawa and his colleagues conducted a study on nonalcoholic liver disease biopsy samples to evaluate the FcγRIIb expression on LSECs. The result showed an inverse proportional relationship between FcγRIIb expression and fibrosis stages, and the highest expressing levels were observed at the incipient stage of fibrosis while the lowest were found at the third stage of fibrosis [15]. In patients with HCC, Geraud et al. discovered that decreased expression of FcγRIIb was more likely to be associated with a more advanced cancer grade. FcγRIIb was not detectable in 63% of the adjacent tissue specimens in a microarray containing HCC tissue samples [16]. Our study showed that in contrast to the control group, the moderate CHB and severe CHB groups showed significantly decreased FcγRIIb expression. This expression also declined with the development of inflammation and fibrosis in CHB patients. Correlation analysis showed FcγRIIb expression levels on LSECs had negative relationships with ALT and AST, which were consistent with serum results. Moreover, there was a positive relationship between FcγRIIb levels and platelet count. Platelets are remarkably related to liver inflammation and fibrosis. Gomez et al. [38] discovered platelet count of patients with nonalcoholic steatohepatitis decreased with the development of liver fibrosis. They also reported that the platelet count could work as a new indirect marker of end-stage liver diseases and portal hypertension evaluation. Our study also showed a negative relationship between inflammation grade and fibrosis stage and FcγRIIb expression levels via regression assay. In other words, patients with CHB who had decreased FcγRIIb expression levels on LESCs may also have a lower platelet count, a higher degree of inflammation and more severe fibrosis. FcγRIIb may be related to enhancements in liver inflammation and fibrosis, and can also be used as indicators to predict inflammation degree and fibrosis stage in CHB patients. However, certain limitations in the present study must be taken into account. First, both the serum sample and liver tissue sizes were not large enough. Additionally, a validation cohort should be evaluated to verify the results. Furthermore, more studies are needed to evaluate the importance and to unveil the function of FcγRIIb in chronic HBV infection and unveil the related function.

## Conclusion

In summary, our research supports the conclusion that FcγRIIb levels are significantly related to chronic HBV infection and progression in CHB. Changes in FcγRIIb may influence the progression of hepatic inflammation and fibrosis in CHB patients.

## Data Availability

The datasets used and/or analyzed during the current study are available from the corresponding author on reasonable request.

## Abbreviations

HBV: Hepatitis B virus
CHB: Chronic hepatitis B
HC: Healthy control
HBeAg: Hepatitis B e antigen
FcγRIIb: Fc gamma receptor IIb
